# Alteration of chondroitin sulfate composition on proteoglycan produced by knock-in mouse embryonic fibroblasts whose versican lacks the A subdomain

**DOI:** 10.1080/03009730902761722

**Published:** 2009-04-24

**Authors:** Keittisak Suwan, Sonoko Hatano, Prachya Kongtawelert, Peraphan Pothacharoen, Hideto Watanabe

**Affiliations:** ^1^Thailand Excellence Center for Tissue Engineering, Department of Biochemistry, Faculty of Medicine, Chiang Mai UniversityChiang MaiThailand; ^2^Institute for Molecular Science of Medicine, Aichi Medical UniversityAichiJapan

**Keywords:** Chondroitin sulfate, extracellular matrix, mouse embryonic fibroblasts, versican

## Abstract

Versican/proteoglycan-mesenchymal (PG-M) is a large chondroitin sulfate (CS) proteoglycan of the extracellular matrix (ECM) that is constitutively expressed in adult tissues such as dermis and blood vessels. It serves as a structural macromolecule of the ECM, while in embryonic tissue it is transiently expressed at high levels and regulates cell adhesion, migration, proliferation, and differentiation. Knock-in mouse embryonic (*Cspg2*^*Δ3/Δ3*^) fibroblasts whose versican lack the A subdomain of the G1 domain exhibit low proliferation rates and acquire senescence. It was suspected that chondroitin sulfate on versican core protein would be altered when the A subdomain was disrupted, so fibroblasts were made from homozygous *Cspg2*^*Δ3/Δ3*^ mouse embryos to investigate the hypothesis. Analysis of the resulting versican deposition demonstrated that the total versican deposited in the *Cspg2*^*Δ3/Δ3*^ fibroblasts culture was approximately 50% of that of the wild type (WT), while the versican deposited in the ECM of *Cspg2*^*Δ3/Δ3*^ fibroblasts culture was 35% of that of the WT, demonstrating the lower capacity of mutant (*Cspg2*^*Δ3/Δ3*^) versican deposited in the ECM. The analysis of CS expression in the *Cspg2*^*Δ3/Δ3*^ fibroblasts culture compared with wild-type fibroblasts showed that the composition of the non-sulfate chondroitin sulfate isomer on the versican core protein increased in the cell layer but decreased in the culture medium. Interestingly, chondroitin sulfate E isomer was found in the culture medium. The amount of CS in the *Cspg2*^*Δ3/Δ3*^ cell layer of fibroblasts with mutant versican was dramatically decreased, contrasted to the amount in the culture medium, which increased. It was concluded that the disruption of the A subdomain of the versican molecule leads to lowering of the amount of versican deposited in the ECM and the alteration of the composition and content of CS on the versican molecule.

## Introduction

Versican/PG-M is a large chondroitin sulfate (CS) proteoglycan of the extracellular matrix (ECM), mainly synthesized by fibroblasts and vascular smooth muscle cells. Its core protein consists of two globular domains G1 and G3 at the N- and C-termini, respectively, and two CS attachment domains, CSα and CSβ, between the two globular domains. As many as 23 CS chains can be attached to these domains, causing the molecular mass of versican to reach up to 1,000 kDa. The N-terminal G1 domain consists of A, B, B′ looped subdomains. The B-B′ stretch binds hyaluronan, and the A subdomain enhances the binding (1). The C-terminal G3 domain binds other ECM molecules, including fibrillins (2), fibulin-1,2 (3,4), tenascin (5–7), type I collagen, and fibronectin (8). By interacting with these molecules, versican is incorporated into the ECM and serves as a structural macromolecule.

Versican exhibits two distinct expression patterns. In adult tissues it is constitutively expressed in epithelial and connective tissues and serves as a structural macromolecule of the ECM, while in embryonic tissue it is transiently expressed at high levels and regulates cell behavior such as adhesion (9–11), migration (12–14), proliferation (15–17), and differentiation (18,19). The G3 domain of versican has been shown to enhance the proliferation of NIH3T3 fibroblasts through an epidermal growth factor (EGF)-like motif (20). The G3 domain without the EGF-like motif enhances the interaction of EGF receptor (EGFR) and β1-integrin, impairing the growth of U87 astrocytoma cells (21). The V1 variant enhances proliferation and inhibits the apoptosis of NIH3T3 fibroblasts, while the V2 variant does the opposite (17). Chondroitin sulfate chains on the versican molecule bind to several biological molecules such as chemokines, CD44, and selectins. Serreno et al. reported that the short V3 isoform of versican that lacks CS chains could reverse the malignant phenotype of melanoma cells (22).

Recently, we generated knock-in mice (*Cspg2*^*Δ3/Δ3*^) whose versican lacks the A subdomain of the G1 domain. These mice, expressing the mutant versican at a level of ~50% (compared with normal versican in wild-type mice), exhibit abnormalities in cardiovascular systems and skin, contrasting to versican-null *hdf* mice which die at E9.5 from severe cardiac defects (23). As *hdf* heterozygotes expressing ~50% normal versican are viable with no obvious phenotype, the abnormalities of embryos are likely due to decreased levels of versican deposition in the ECM by the absence of the A subdomain.

Embryonic fibroblasts obtained from *Cspg2*^*▵3/▵3*^ mice exhibited unusual characteristics. Within 20 passages, the rate of proliferation of the *Cspg2*^*▵3/▵3*^ fibroblasts slowed, and the cells exhibited senescence. Whereas the extracellular matrix of the wild-type fibroblasts exhibited a network structure of hyaluronan and versican, that of the *Cspg2*^*▵3/▵3*^ fibroblasts exhibited~30% and~85% deposition of versican and hyaluronan (HA) without such a structure. In this experiment we examined the chondroitin sulfate composition in proteoglycan molecules from *Cspg2*^*▵3/▵3*^ fibroblasts culture comparing them with wild-type (WT) fibroblasts culture.

## Materials and methods

### WT and the Cspg2^▵3/▵3^ mouse embryonic fibroblast culture preparation

Female *Cspg2*^*WT/▵3*^ and male *Cspg2*^*WT/▵3*^ mice were mated and allowed to gestate. These mice produced WT heterozygous (*Cspg2*^*WT/▵3*^) and homozygous (*Cspg2*^*▵3/▵3*^) offspring whose versican lacked the A subdomain. After 12 days, the pregnant mice were killed using carbon dioxide gas. The placentas were removed, and each embryo was separated and placed in a Petri dish containing 1% penicillin/streptomycin phosphate buffer saline (PBS) solution. Each embryo's tail was cut and collected in a microcentrifuge tube for further genotyping. The rest of the embryo was roughly cut into small pieces using scissors. Trypsin/EDTA was added to those pieces, and then incubated for 15–30 minutes. Dulbecco's modified Eagle's medium (DMEM) containing 10% fetal bovine serum (FBS) and 1% penicillin/streptomycin was added to stop the reaction. The medium was removed by suction, and the remaining tissue was passed through a cell strainer to get single cells. Medium was then added, and the cells were cultured until they reached confluence. At the first and second passages, fibroblasts in the culture were collected and stored in liquid nitrogen for future use.

### Mouse embryo genotyping

Each mouse embryo tail was soaked in proteinase K overnight. Genomic DNA was then extracted using a DNeasy kit (Qiagen). The extracted DNA was analyzed by the genomic polymerase chain reaction (PCR) method using PCR mixtures for genomic PCR composed of 10X Extaq buffer, 2.5 mM dNTP , deionized water, Extaq polymerase, and primers for WT/mutant allele. To detect the mutant allele, a sense primer, 5?-CTGAAGAAGGAGATCAGCAGCCTC-3?, and an antisense primer, 5?-GTGTGGGTTCCAGGAATCGTACTGAG-3?, were used. To detect the WT allele, a sense primer, 5?-ATTAATTCTAGACTGGAACTTAGAGC-3?, and an antisense primer, 5?-TCTTACAGATCTGAATGCCTTACCATCC-3?, were used. Exactly 9 µL of PCR mixture was aliquoted to a PCR tube, then 1 µL of embryo DNA was added. The PCR reaction test for WT and mutant alleles was performed. The PCR products were applied to 1% agarose gel, and electrophoresis was performed. Visual bands, produced using ethidium bromide, were used to identify embryo alleles.

### [^35^S] incorporation assay

Semi-confluent mouse embryonic fibroblasts were labeled with [^35^S]-sulfate (100 µCi/mL) in 100 mm dishes for 24 hours. The medium and cell layer were collected separately and extracted using 4 M Guanidinium Hydrochloride (GuHCl) as previously described (24). Proteoglycans were precipitated with 3 volumes of 95% (v/v) ethanol containing 1.3% (w/v) potassium acetate. The precipitate was dissolved in buffer A (7 M urea, 50 mM Tris-HCl pH 7.5, 0.01% Triton X) and then rotated at 4°C overnight. The sample was applied to a DEAE-Sephacel column. Eluate fractions by 2 M NaCl were collected and combined. The eluted sample was applied to a gel filtration (Superose6) column. The proteoglycan fraction was collected, and its radioactivity was measured using a Beckman Coulter^TM^ LS6500 multi-purpose scintillation counter.

### Chondroitin sulfate composition analysis

Fibroblasts were cultured in 60 mm culture dishes up to confluence, after which the medium and cells were collected. Cell lysate, prepared using Cytobuster reagent (Novagens), was centrifuged at 13,000 *g* at 4°C for 10 minutes, after which the supernatant was collected. A sample of supernatant and culture medium were each applied to a separate 0.3 mL DEAE-Sephacel column and equilibrated with the equilibration buffer (50 mM Tris-HCl pH 7.2, 0.1 M NaCl). After washing with 3 mL of the equilibration buffer, the proteoglycan fraction was eluted with elution buffer (50 mM Tris-HCl pH 7.2, 2 M NaCl). Three volumes of 95% ethanol containing 1.3% potassium acetate were added to the eluate. The solution was chilled at −20°C overnight and then centrifuged at 13,000 *g* for 30 minutes at 4°C. The resulting pellet of proteoglycan was washed twice with 98% ethanol and then dried. After the pellet was reconstituted in deionized water, the protein concentration in the precipitated pellet of proteoglycan was determined by bicinchoninic acid (BCA) assay. Equal amounts of extracted proteoglycan from WT and *Cspg2*^*Δ3/Δ3*^ fibroblasts were dissolved in chondroitinase ABC buffer (20 mM Tris-HCl pH 8.0, 20 mM sodium acetate, 0.02% bovine serum albumin (BSA)) and digested with chondroitinase ABC (5 mU/mL) at 37°C for 3 hours. The reaction was stopped by boiling at 100°C for 5 minutes.

The resulting solution was centrifuged at 14,000 rpm for 15 minutes. (Prior to centrifuging the solution, the centrifuge was prepared by adding 100 µL of distilled water (high performance liquid chromatography grade) to the centrifugal filter and centrifuging it twice for 4 minutes each at 14,000 rpm, removing the flow-through each time.) The flow-through was collected for further analysis by high performance liquid chromatography (HPLC). The sample was added to a HPLC vial tube, and a postcolumn reaction HPLC was performed. The unsaturated CS disaccharides product was analyzed by fluorometric postcolumn HPLC, and its content was calculated from the standard curve of chondroitin sulfate-derived disaccharides.

### Protein assay

The bicinchoninic acid (BCA) assay procedure (Pierce) was used to determine the concentration of protein in the sample. First, standard albumin was prepared to establish a calibration curve (2 mg/mL bovine serum albumin was diluted to get concentration ranges from 0.5 to 200 µg/mL). Then reagent A (sodium carbonate, sodium bicarbonate, and sodium tartrate in 0.2 M sodium hydroxide), reagent B (4% bicinchoninic acid), and reagent C (4% cupric sulfate pentahydrate) were thoroughly mixed at the ratio of 50:48:2, respectively, to make the working reagent. Each 25 µL of the standard or unknown sample was placed on a microplate and combined with 200 µL of the BCA working reagent. The combination was mixed thoroughly on the plate shaker for 30 seconds. The plate was then covered and incubated at 37°C for 30 minutes, then cooled and measured at absorbance 562 nm on the plate reader. The sample concentration was calculated from the calibration curve of standard bovine serum albumin.

## Results

### Genotyping of mouse embryos

**Figure 1.**
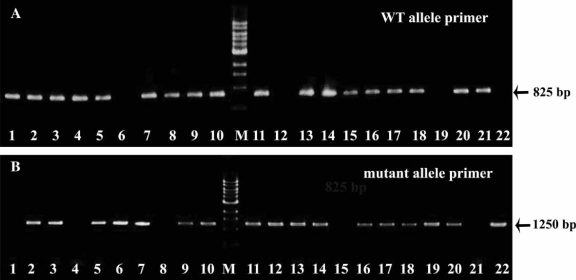
Agarose gel electrophoresis of PCR products from mouse embryo tails. A: wild-type allele determination by genomic PCR; B: mutant allele determination by genomic PCR. The numbers in figures A and B represent embryo as follows: number 1 = embryo no. 1; number 2 = embryo no. 2; number 3 = embryo no. 3; number 4 = embryo no. 4; number 5 = embryo no. 5; number 6 = embryo no. 6; number 7 = embryo no. 7; number 8 = embryo no. 8; number 9 = embryo no. 9; number 10 = embryo no. 10; M = 1 kb DNA ladder; number 11 = embryo no. 11; number 12 = embryo no. 12; number 13 = embryo no. 13; number 14 = embryo no. 14; number 15 = embryo no. 15; number 16 = embryo no. 16; number 17 = embryo no. 17; number 18 = embryo no. 18; number 19 = embryo no. 19; number 20 = embryo no. 20; number 21 = embryo no. 21; number 22 = embryo no. 22.

To characterize the genotypes of the mouse embryos, 22 embryos were isolated from heterozygous pregnant *Cspg2*^*WT/Δ3*^ mice, embryo tails were collected, and genomic DNA was extracted. The genotype of each embryo was characterized by using the genomic PCR technique. Two primer sets were used for genotype characterization including WT, heterozygous mutant, and homozygous mutant alleles. The PCR products from the WT allele revealed a band for the WT primers set (Figure 1). The PCR products from the homozygous mutant allele revealed a band for the mutant primers set. The PCR products from the heterozygous mutant allele revealed bands for both the WT and mutant primers sets. The results indicated that embryos number 1, 4, 8, 15, and 21 were WT allele, embryos number 6, 12, 19, and 22 were homozygous allele, and the remaining embryos were heterozygous allele. These results were used for choosing fibroblast culture to perform further experiments.

**Figure 2.**
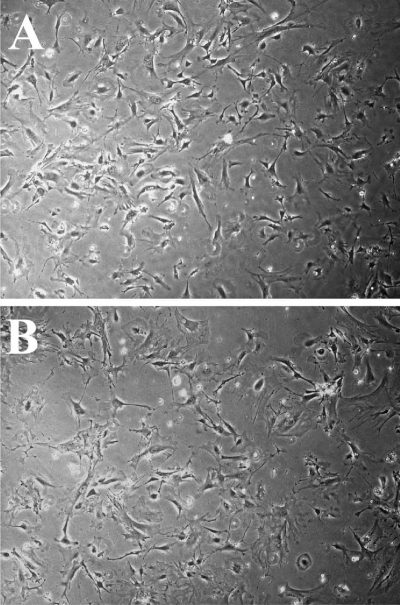
Representative photographs (4× magnification) showing the cell morphology of wild-type (WT) fibroblasts (A) and *Cspg2*^*Δ3/Δ3*^ fibroblasts (B) at passage no. 4.

We observed that the heterozygous *Cspg2*^*WT/Δ3*^ mice were viable, fertile, and without any obvious abnormalities like WT mice, but the *Cspg2*^*Δ3/Δ3*^ homozygotes died at the embryonic stage. Therefore, to study the abnormalities of *Cspg2*^*Δ3/Δ3*^ homozygotes, homozygote alleles (*Cspg2*^*Δ3/Δ3*^ and *Cspg2*^*WT/WT*^) were chosen to make fibroblast cultures. The morphology of WT fibroblasts differed from that of the *Cspg2*^*Δ3/Δ3*^ fibroblasts. The *Cspg2*^*Δ3/Δ3*^ fibroblasts appeared larger, flatter, and more spread out than WT fibroblasts (Figure 2). In previous experiments, we found that the proliferation rate of the *Cspg2*^*Δ3/Δ3*^ fibroblasts was slower than that of the WT fibroblasts, and that the versican expression level was reduced in the *Cspg2*^*Δ3/Δ3*^ fibroblast cultures. *In vitro* studies done by Miquel-Serra et al. (25) on the expression of versican isoform lacking the chondroitin sulfate chain showed negative effects on primary cell growth, similar to our results. Therefore, we suspected that the characteristics of the chondroitin sulfate on the versican molecules of *Cspg2*^*Δ3/Δ3*^ fibroblasts might explain some of the differences shown. For our next experiment, we determined the chondroitin sulfate composition in versican extracted from *Cspg2*^*Δ3/Δ3*^ fibroblasts and compared it with WT versican.

### Decrease of chondroitin sulfate proteoglycan deposition in Cspg2^Δ3/Δ3^ fibroblasts cultures

To measure the deposition of chondroitin sulfate proteoglycan in the *Cspg2*^*Δ3/Δ3*^ fibroblast cultures, semi-confluent fibroblasts were cultured overnight in the medium containing [^35^S]-sulfate because cells will consume [^35^S]-sulfate to be a substrate for chondroitin sulfate synthesis, and [^35^S]-labeled chondroitin sulfate will be assembled with proteoglycan core protein. Chondroitin sulfate proteoglycan was extracted from the culture medium and cell layer of [^35^S]-sulfate-treated fibroblast cultures. The amounts of proteoglycan deposited in the cell layer and medium were indicated by the amount of [^35^S] detected by the scintillation counter. Since versican (23 CS chains bearing chondroitin sulfate proteoglycan) is a major chondroitin sulfate proteoglycan expressed in the fibroblast cultures (26), we expected that the extracted chondroitin sulfate proteoglycan from mouse embryonic fibroblast cultures in this experiment would represent as versican. However, fibroblasts are also able to produce decorin (27), a CS chain bearing chondroitin sulfate proteoglycan. To verify whether the mouse embryonic fibroblasts cultures in this study produce only versican molecules, Western blot analysis of versican and decorin molecules was performed by using antibodies against versican and decorin molecules, respectively. The results revealed that only versican was detected in the fibroblast cultures (data not shown). We can conclude, therefore, that mouse embryonic fibroblasts in the study produce only versican as chondroitin sulfate proteoglycan.

**Figure 3.**
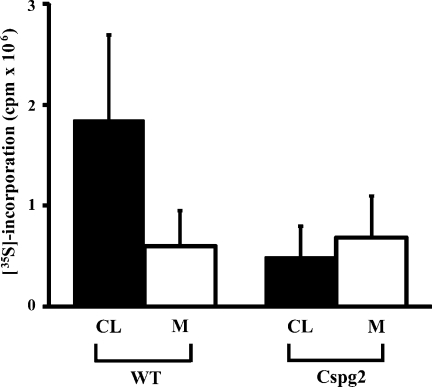
Versican deposition in WT and *Cspg2*^*Δ3/Δ3*^ fibroblast cultures. The deposition of versican molecules was detected by the [^35^S] incorporation assay; the amount of versican deposition was represented as the intensity of [^35^S] isotope (cpm×10^6^). The closed bar (CL) represents the amount of versican deposited in cell layer, and the open bar (M) represents the amount of versican deposited in the culture medium. Data are the mean value±standard deviation of three independent experiments. WT = wild-type fibroblasts; *Cspg2*^*Δ3/Δ3*^=*Cspg2*^*Δ3/Δ3*^ (mutant) fibroblasts.

[^35^S]-sulfate incorporation studies revealed that the total versican (culture medium and cell layer) secreted by *Cspg2*^*Δ3/Δ3*^ fibroblasts was approximately 50% of the total secreted by WT (Figure 3). Of the total secreted versican of the WT, 70% was deposited in the cell layer, while only 35% of the total secreted versican of the *Cspg2*^*Δ3/Δ3*^ was deposited in the cell layer. This result clearly demonstrated the lower capacity of mutant (*Cspg2*^*Δ3/Δ3*^) fibroblasts to deposit versican in the cell layer.

### Chondroitin sulfate composition on the versican molecule alters in the Cspg2^Δ3/Δ3^ fibroblast cultures

To analyze the chondroitin sulfate pattern on the versican molecule, fibroblasts were cultured until they reached confluence. Proteoglycan was then extracted from both the culture medium and cell layer. Equal amounts of proteoglycan from WT and mutant fibroblasts were deposited in separate reaction tubes. The chondroitin sulfate chains on the versican core protein were digested into disaccharide units by chondroitinase ABC. HPLC was used to determine the chondroitin sulfate content and composition.

The chondroitin sulfate composition on the versican core protein was analyzed. The cell layer of the WT fibroblast cultures contained chondroitin 4-sulfate as the major isomer (87%), while the non-sulfate chondroitin sulfate was found as the minor (13%) isomer as shown in Table I. The cell layer of the *Cspg2*^*Δ3/Δ3*^ fibroblasts culture consisted of 81% chondroitin 4-sulfate and 19% non-sulfate chondroitin sulfate. Likewise, the culture medium of WT fibroblasts contained chondroitin 4-sulfate as the major isomer (59%), whereas non-sulfate chondroitin sulfate (33%) and chondroitin 6-sulfate (8%) were the minor isomers. The culture medium of *Cspg2*^*Δ3/Δ3*^ fibroblasts contained chondroitin 4-sulfate (59%), non-sulfate chondroitin sulfate (28%), and chondroitin 6-sulfate (12%). Remarkably, a trace amount (1%) of chondroitin sulfate E was also found. Thus, our results showed that the percentage of the chondroitin 4-sulfate isomer in the culture medium of *Cspg2*^*Δ3/Δ3*^ fibroblasts was the same as that in the culture medium of WT fibroblasts, but the percentage of the non-sulfate chondroitin sulfate tended to be lower than that of the culture medium of WT fibroblasts. We also found that the negative charge of the chondroitin sulfate isomers in the culture medium of *Cspg2*^*Δ3/Δ3*^ fibroblasts appeared to be higher than that of the culture medium of WT fibroblasts.

**Table I.** Chondroitin sulfate composition in cell layer and fibroblast culture medium; data shown are the percent content of chondroitin sulfate. Data are the mean values of three independent experiments±standard deviation.

	% Chondroitin sulfate content			
	Cell layer		Medium	
	Wild type	Cspg2	Wild type	Cspg2
Δdi0S	13±0.11	19±0.79	33±0.50	28±0.30
Δdi4S	87±0.11	81±0.11	59±0.55	59±0.22
Δdi6S	ND	ND	8±0.05	12±0.33
ΔdiSE	ND	ND	ND	1±0.09
ΔdiSD	ND	ND	ND	ND
ΔdiTriS	ND	ND	ND	ND

Δdi0S: non-sulfate chondroitin sulfate, Δdi4S: chondroitin 4-sulfate, Δdi6S: chondroitin 6-sulfate, ΔdiSE: chondroitin 4,6-sulfate, ΔdiSD:chondroitin 2,6-sulfate, ΔdiTriS: chondroitin 2,4,6-sulfate.

**Figure 4.**
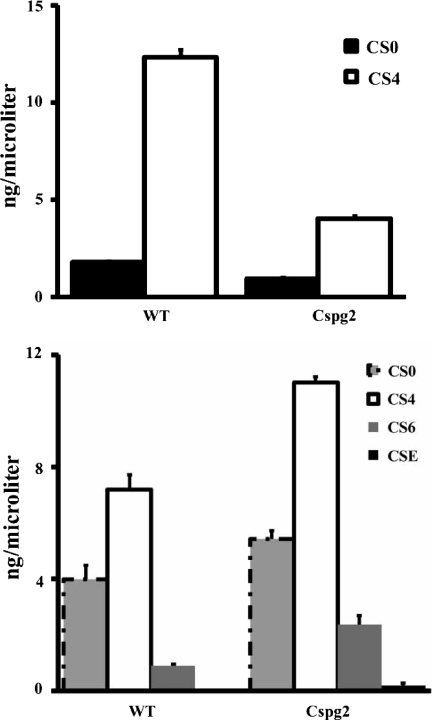
Chondroitin sulfate content on versican core protein which deposited in cell layer (upper graph) and culture medium (lower graph) of fibroblasts using high performance liquid chromatography (HPLC) analysis. Data are the mean values of three independent experiments±standard deviation. Each bar represents the chondroitin sulfate isomer.

We found that the cell layer of the WT fibroblasts culture contained 14.1 ng/µL of chondroitin sulfate from the versican molecule, while the cell layer of the *Cspg2*^*Δ3/Δ3*^ fibroblast cultures contained 5.0 ng/µL of chondroitin sulfate, nearly three times less (Figure 4). The WT fibroblast culture medium contained 12.1 ng/µL of chondroitin sulfate on the versican molecule, while the *Cspg2*^*Δ3/Δ3*^ fibroblast culture medium contained 19.0 ng/µL, one-and-a-half times more (Figure 4). These data showed that the amount of chondroitin sulfate on the mutant versican molecule in the cell layer was dramatically lower than that in the WT cell layer, while the amount in the culture medium was higher than in the WT medium.

We also found that the cell layer of the WT fibroblasts culture contained 12.3 ng/µL of chondroitin 4-sulfate on the versican molecule, while the cell layer of the *Cspg2*^*Δ3/Δ3*^ fibroblast cultures contained 4.0 ng/µL, three times less. This demonstrates that versican molecules in *Cspg2*^*Δ3/Δ3*^ fibroblast cultures contain less negatively charged chondroitin sulfate in the extracellular matrix. Interestingly, the negatively charged chondroitin sulfate increased in the *Cspg2*^*Δ3/Δ3*^ fibroblast culture medium. We concluded that the disruption of the A subdomain of the versican molecule leads to the alteration of the composition and content of chondroitin sulfate on the versican molecule.

## Discussion

In this study we compared certain characteristics of fibroblasts from *Cspg2*^*Δ3/Δ3*^ mouse embryos with those of WT mouse embryos, which were studied earlier. The mutant cells had a slower growth rate and appeared larger and more spread out than cells from WT fibroblasts. Yang et al. (28) reported that the presence of the G1 domain inhibits chondroitin sulfate chain attachment to versican core protein. Since the A subdomain is part of the G1 domain, we suspected that chondroitin sulfate composition and content on the versican core protein would be changed if the A subdomain was disrupted. We investigated this hypothesis by using the *Cspg2*^*Δ3/Δ3*^ fibroblasts as a model. It was found that versican deposition in the *Cspg2*^*Δ3/Δ3*^ fibroblast cultures was approximately 50% less than the versican deposition in the WT fibroblast cultures. A recent study of versican mRNA in our laboratory showed a decrease in versican expression in the *Cspg2*^*Δ3/Δ3*^ fibroblast cultures (unpublished observations). Those results suggested that loss of the A subdomain affected the expression of proteoglycan in mutant fibroblasts. The experiment discussed in this study found that the versican deposition in the ECM of *Cspg2*^*Δ3/Δ3*^ fibroblasts culture was approximately 35% of that in the ECM of WT fibroblasts culture, while the deposition of versican in the medium of *Cspg2*^*Δ3/Δ3*^ fibroblast cultures tended to be higher than in the culture medium of WT fibroblasts. Matsumoto et al. showed that versican binds hyaluronan at the B-B′ stretch of the G1 domain and that the A subdomain enhances their interaction up to 17-fold (1).

Our analysis of chondroitin sulfate was as we suspected. When the A subdomain was disrupted, chondroitin sulfate composition on the versican core protein was changed. Non-sulfate chondroitin sulfate isomer tended to increase in the cell layer but to decrease in the culture medium. This demonstrated that the negative charge on versican molecules decreased in the ECM but increased in the culture medium. Interestingly we found chondroitin sulfate E (di-sulfated chondroitin sulfate) on the versican molecule in the culture medium. The chondroitin sulfate chain has a negative charge which makes it possible for proteoglycans to bind various positively charged molecules such as growth factors, cytokines, and chemokines (29). Moreover it allows proteoglycans in the ECM or on the cell surface to generate immobilized gradients of certain growth factors and present them to their receptors; chondroitin sulfate chains also protect proteoglycans from proteolytic degradation (30). We found that both the charge and the content of chondroitin sulfate on the versican molecule were decreased in the cell layer of the *Cspg2*^*Δ3/Δ3*^ fibroblast cultures. Other studies have shown that the charge of chondroitin sulfate regulates cell activity. For example, the number of neurites decreased with high di-sulfation chondroitin sulfate (31), and chondroitin sulfate tetrasaccharide stimulated the growth and differentiation of neurons (32).

Fluctuation in chondroitin sulfate content has been found to affect biological activity. An increase in chondroitin sulfate chains in cephalic neural crest migratory routes not only disrupts their migration, but also impedes detachment of the rhombencephalic neuroepithelium neural crest cells (33). Evidence suggests, then, that the decrease of the total charge and deposition of versican molecules in the *Cspg2*^*Δ3/Δ3*^ fibroblasts cell layer will affect the proliferation and morphology of fibroblasts. We can conclude, then, that the *Cspg2*^*Δ3/Δ3*^ mutant versican molecule, which contains a lower negative charge and less chondroitin sulfate on its core protein than normal versican, does not maintain growth factors and provides other essential molecules to serve cells, and thus leads to the alteration of cell behavior, such as a slow growth rate and the acquisition of senescence in the *Cspg2*^*Δ3/Δ3*^ fibroblasts.

In addition, Matsumoto et al. (34) showed that the chondroitin sulfate composition on the versican molecule of WT mouse embryo fibroblasts differed from the composition in mouse cartilage. They found more non-sulfated chondroitin sulfate than chondroitin 4-sulfate in mouse cartilage, but more chondroitin 4-sulfate than non-sulfated chondroitin in mouse fibroblasts. This is more evidence that, even though versican is distributed throughout the body, the composition of chondroitin sulfate on the versican molecule is different for different tissues.
